# Diagnosis and segmentation effect of the ME-NBI-based deep learning model on gastric neoplasms in patients with suspected superficial lesions - a multicenter study

**DOI:** 10.3389/fonc.2022.1075578

**Published:** 2023-01-16

**Authors:** Leheng Liu, Zhixia Dong, Jinnian Cheng, Xiongzhu Bu, Kaili Qiu, Chuan Yang, Jing Wang, Wenlu Niu, Xiaowan Wu, Jingxian Xu, Tiancheng Mao, Lungen Lu, Xinjian Wan, Hui Zhou

**Affiliations:** ^1^ Department of Gastroenterology, Shanghai General Hospital, Shanghai Jiao Tong University School of Medicine, Shanghai, China; ^2^ Shanghai Key Laboratory of Pancreatic Diseases, Shanghai Jiao Tong University School of Medicine, Shanghai, China; ^3^ Department of Gastroenterology, Shanghai Jiao Tong University Affiliated Sixth People’s Hospital, Shanghai, China; ^4^ Department of Gastroenterology, Shanghai Tong Ren Hospital, Shanghai Jiao Tong University School of Medicine, Shanghai, China; ^5^ School of Mechanical Engineering, Nanjing University of Science and Technology, Nanjing, China; ^6^ Department of Pathology, Shanghai General Hospital, Shanghai Jiao Tong University School of Medicine, Shanghai, China

**Keywords:** deep learning, suspected superficial lesions, magnifying endoscopy with narrow band imaging (ME-NBI), gastric neoplastic lesions, convolutional neural network (CNN)

## Abstract

**Background:**

Endoscopically visible gastric neoplastic lesions (GNLs), including early gastric cancer and intraepithelial neoplasia, should be accurately diagnosed and promptly treated. However, a high rate of missed diagnosis of GNLs contributes to the potential risk of the progression of gastric cancer. The aim of this study was to develop a deep learning-based computer-aided diagnosis (CAD) system for the diagnosis and segmentation of GNLs under magnifying endoscopy with narrow-band imaging (ME-NBI) in patients with suspected superficial lesions.

**Methods:**

ME-NBI images of patients with GNLs in two centers were retrospectively analysed. Two convolutional neural network (CNN) modules were developed and trained on these images. CNN1 was trained to diagnose GNLs, and CNN2 was trained for segmentation. An additional internal test set and an external test set from another center were used to evaluate the diagnosis and segmentation performance.

**Results:**

CNN1 showed a diagnostic performance with an accuracy, sensitivity, specificity, positive predictive value (PPV) and negative predictive value (NPV) of 90.8%, 92.5%, 89.0%, 89.4% and 92.2%, respectively, and an area under the curve (AUC) of 0.928 in the internal test set. With CNN1 assistance, all endoscopists had a higher accuracy than for an independent diagnosis. The average intersection over union (IOU) between CNN2 and the ground truth was 0.5837, with a precision, recall and the Dice coefficient of 0.776, 0.983 and 0.867, respectively.

**Conclusions:**

This CAD system can be used as an auxiliary tool to diagnose and segment GNLs, assisting endoscopists in more accurately diagnosing GNLs and delineating their extent to improve the positive rate of lesion biopsy and ensure the integrity of endoscopic resection.

## 1 Introduction

Gastric cancer is one of the most prevalent malignant tumours with high morbidity and mortality worldwide (1). Early diagnosis and appropriate treatment are key measures in reducing the mortality rate of gastric cancer; however, most patients are diagnosed at a late stage. Intestinal-type gastric cancer develops through a “Correa cascade”: chronic atrophic gastritis, intestinal metaplasia, and gastric intraepithelial neoplasia (GIN) (2). GIN is the final stage of gastric carcinogenesis and is defined as intraepithelial neoplasia with no clear evidence of depth invasion (3). The newest Western guideline recommends that endoscopically visible low-grade intraepithelial neoplasia (LGIN) lesions should undergo endoscopic resection, similar to high-grade intraepithelial neoplasia (HGIN) and early gastric cancer (EGC), because of a high rate of histological upstaging after resection (4). Therefore, accurate diagnosis and prompt treatment of gastric neoplastic lesions (GNLs) including EGC and GIN, is necessary in clinical practice.

The application of magnifying endoscopy with narrow-band imaging (ME-NBI) dramatically improves the detection rate of EGC and its precancerous lesions compared to white light endoscopy (WLE) (5). According to Yao’s research, EGC can be diagnosed under ME-NBI if the lesion has a demarcation line (DL) and an irregular microsurface (MS) pattern and/or an irregular microvessel (MV) pattern, which is called the “vascular plus surface classification system (VSCS)” (6). However, in clinical practice, it is not easy for endoscopists to judge whether the MS or MV of some atypical superficial gastric lesions is regular, which depends on the experience and knowledge reserve of endoscopists with EGC. At present, there is no accepted standard for the endoscopic diagnosis of LGIN. Previous studies have shown that MS and MV are often regular in LGIN lesions, while DL sometimes exists because of the differences in VS morphology between lesion mucosa and background mucosa (7). In our previous study, an auxiliary index with a high accuracy of identifying LGIN under ME-NBI was proposed – endoscopic acanthosis nigricans appearance (EANA) (8). For LGIN lesions of type 0-IIa, studies have shown that the morphological characteristics of dense-type crypt opening or regular white opaque substance (WOS) under ME-NBI can be helpful in distinguishing them from carcinomas (9, 10). Some endoscopists also use the morphologic evolution of gastric pits based on the Sakaki classification system to identify precancerous lesions and EGC (11). However, the diagnosis of EGC and LGIN under endoscopy requires extensive experience and clinical practice, which is lacking for many endoscopists.

In recent years, with the development of deep learning, artificial intelligence (AI) has been increasingly used in medical image processing and has achieved excellent performance in recognising lesions under upper or lower gastrointestinal endoscopy in different modes, such as WLE, ME-NBI or blue laser imaging (12). Many computer-aided diagnostic (CAD) systems have been established for identifying lesions such as esophageal squamous cell carcinoma, EGC and intestinal metaplasia (13–17). However, most of the previous CAD systems were developed for the diagnosis of EGC, and there is no diagnostic system for GNLs (including LGIN and EGC). In addition, most CAD systems are used for targeting lesions, and few can accurately mark the boundary of the lesions at the same time. Due to the clinical requirements of follow-up endoscopic or surgical resection of GNLs, the extent of lesions should be accurately determined under image-enhanced endoscopy in many cases. Therefore, we developed a CAD system based on deep learning to diagnose and segment GNLs in patients with suspected superficial lesions.

## 2 Materials and methods

### 2.1 Study design and image capture

This retrospective study was performed at three endoscopy centers, located in Shanghai General Hospital – South (center 1), Shanghai Jiao Tong University Affiliated Sixth People’s Hospital (center 2) and Shanghai General Hospital – North (center 3). It should be noted that the center 1 and the center 3 are independently operated in different locations, with fixed staff and endoscopy equipment purchased in different years. This study was conducted in accordance with the Declaration of Helsinki and was approved by the Ethics Committee of the Shanghai General Hospital (2020KY236). ME-NBI images from October 2014 to June 2021 were retrospectively collected. Every patient underwent conventional upper gastrointestinal WLE, and then the suspected superficial lesions were carefully examined under ME-NBI. The ME-NBI images were captured by standard endoscopes (GIF-H260Z or GIF-H290Z, Olympus Co., Tokyo, Japan) with an EVIS LUCERA ELITE endoscopic system (CV-290, Olympus Co.) and an endoscopic cold light source (CLV-290SL, Olympus Co.). A black soft hood was attached to the tip of the endoscope to obtain stable images at maximum magnification.

### 2.2 Setting up the datasets

Suspected superficial lesions were confirmed by pathological data obtained from the targeted biopsy samples, endoscopic submucosal dissection (ESD) samples or surgical samples. All pathological data from the three centers were assessed by senior gastrointestinal pathologists according to the Vienna classification (18). Category 3 was defined as LGIN lesions, and Categories 4 and 5 were defined as EGC lesions. LGIN and EGC lesions were defined as GNLs.

To distinguish neoplastic lesions from their background mucosa, ME-NBI images of chronic gastritis and intestinal metaplasia were obtained as background training under the same imaging conditions. Images of esophageal lesions, duodenal lesions, submucosal tumours, those with a lack of pathological data, and images of poor quality (including bleeding, mucous adherence, presence of foreign bodies and out of focus) were excluded from the analysis.

In the ME-NBI images, the following two criteria should be met for labelling superficial gastric lesions as LGIN: (1) there are DLs that can be identified under ME-NBI; (2) the presence of EANA or types IV–VI pit pattern of the Sakaki classification or regular white opaque substance (WOS) or dense-type crypt opening (8–11). The following criteria should be met for labelling superficial gastric lesions as EGC: (1) an irregular MV pattern with DL and (or) (2) an irregular MS pattern with DL (6).

**Figure 1:**
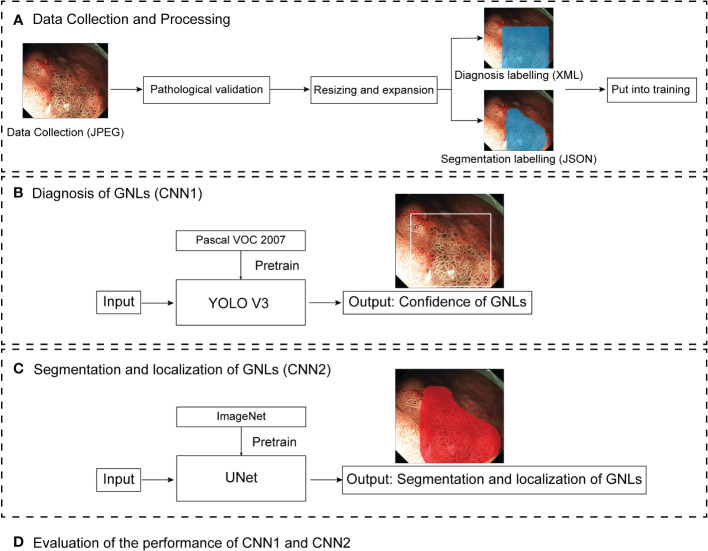
Pipeline diagram of this study. ME-NBI images were collected as JPEG files. After pathological validation, the images were resized and expanded, and labelled by endoscopists for training the different deep learning models. CNN1 and CNN2 are two parallel models. CNN1 output the confidence of GNLs and CNN2 output the segmentation and localisation of GNLs. Finally, the performance of CNN1 and CNN2 were evaluated. ME-MBI, magnifying endoscopy with narrow band imaging; CNN, convolutional neural network; GNLs, gastric neoplastic lesions.

All images were JPEG files with various sizes such as 1020×764 pixels, 716×512 pixels and 764×572 pixels. Baseline data such as the date of diagnosis of GNLs, lesion locations, macroscopic types and maximum diameter, were collected. Then, the redundant parts of the images, such as the patient information and acquisition time, were cropped before labelling. Two senior endoscopists separately labelled the images as “GNLs” or “non-GNLs” according to the pathological data and marked the location of GNLs using rectangular box annotation by LabelImg (version 1.8.6, https://github.com/heartexlabs/labelImg), then saved as XML files. If they were in agreement, the image was considered to be acceptable. If they had any disagreement, then another senior endoscopist made the judgement. An expert endoscopist used polygon annotation to label the extent of the GNLs by Labelme (version 4.2.10, https://github.com/wkentaro/labelme), saved as a JSON file. Before training, the images were resized to 416×416 pixels, and the number of ME-NBI images in the training set was expanded by rotating and flipping. **Figure 1** shows a pipeline diagram of this study.

All of the images were divided into the following datasets:

1. A total of 3757 images from 392 patients with GNLs and 2420 images from 568 patients with non-GNLs from center 1 and center 2 were used to train and test the performance of CNN1 and CNN2. Patients were randomly assigned to the training group and the internal test group, according to the 85:15 ratio of patients with GNLs. The training set consisted of 3331 ME-NBI images of GNLs from 333 patients and 2220 images from 492 patients with non-GNLs from center 1 and center 2, which were used to train CNN1 and CNN2.
2. The internal test set consisted of 200 images from 59 patients with GNLs and 200 images from 76 patients with non-GNLs from center 1 and center 2, which were not involved in the training of the modules and were used to test the performance of CNN1, and another 226 high-definition GNLs images with sufficient magnification from the same 59 patients with GNLs were selected to test the segmentation performance of CNN2.
3. The external test set consisted of 800 images from 106 patients with GNLs and 800 images from 130 patients with non-GNLs. These images were from center 3 and were used to test the generalisation ability of CNN1.
4. The comparison test set consisted of 100 randomly picked GNLs images and 100 non-GNLs images from both the internal and external test sets, which were used to compare the diagnostic performance of CNN1 with that of the endoscopists.

**Figure 2:**
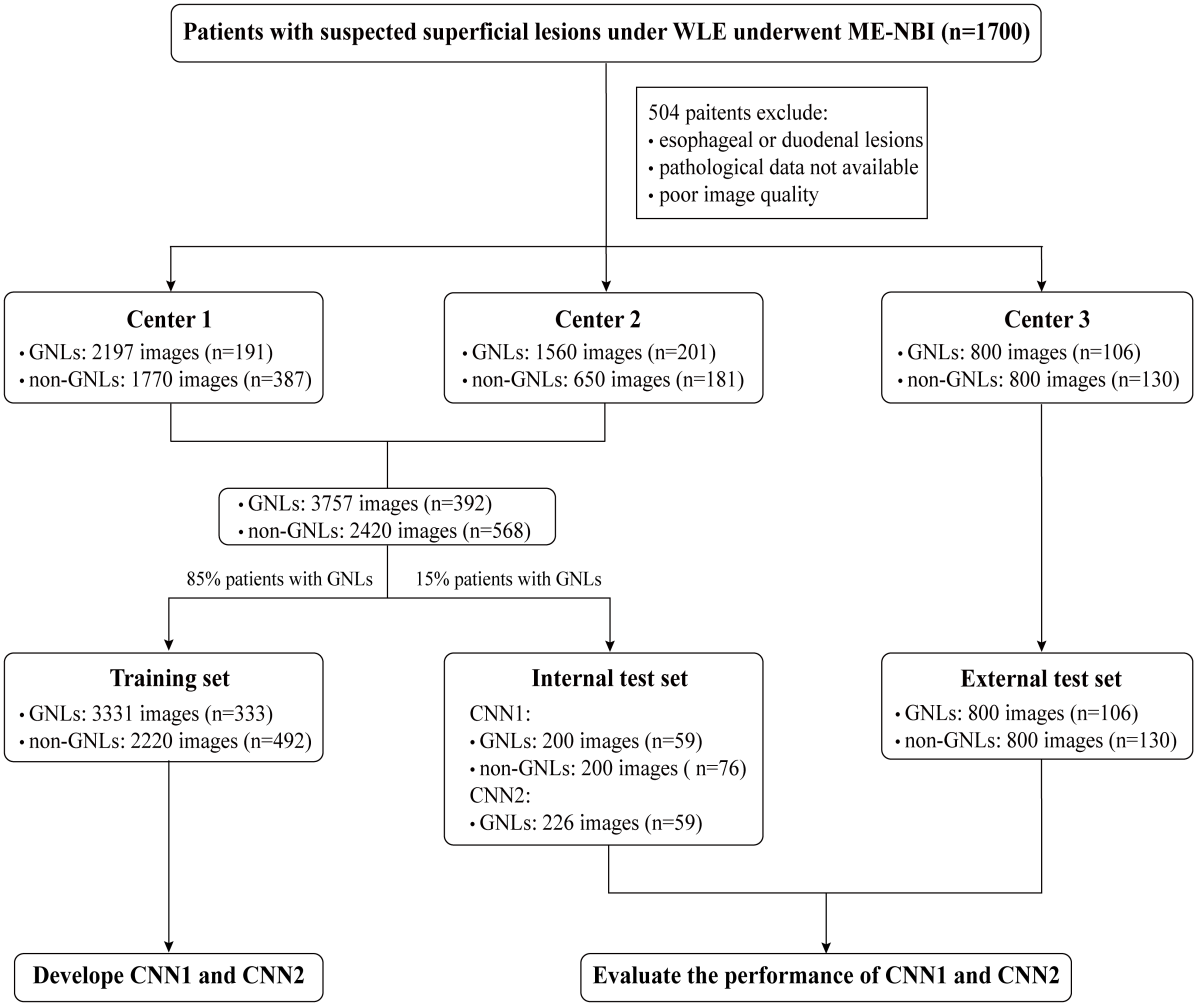
Flowchart of setting the datasets. WLE, white light endoscopy; ME-MBI, magnifying endoscopy with narrow band imaging; CNN, convolutional neural network; GNLs, gastric neoplastic lesions.

A flowchart of setting the datasets is shown in **Figure 2**.

### 2.3 Development of deep learning algorithm

**Figure 3:**
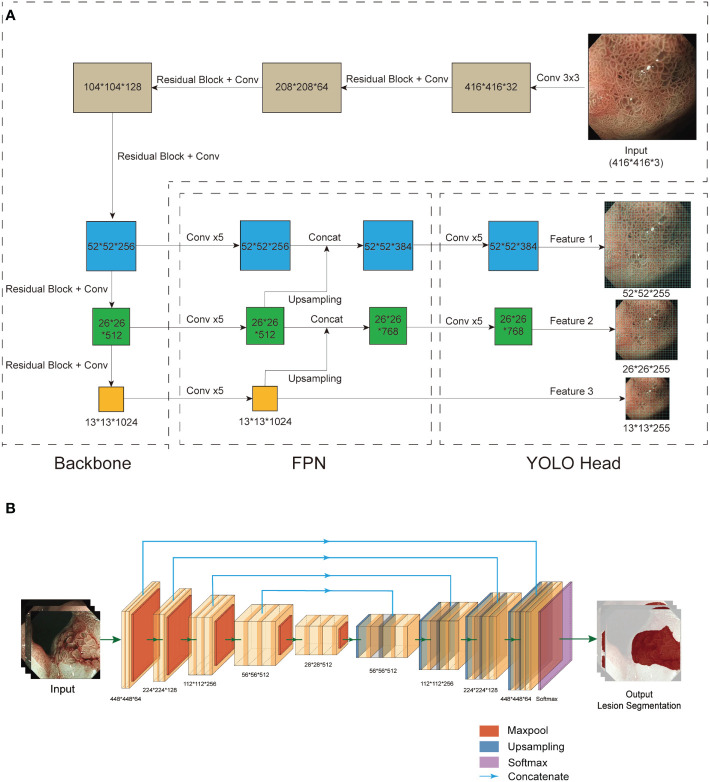
Structure of CNN1 and CNN2. **(A)** CNN1 is based on the YOLO v3 network. Based on the results of the pre-experiment, we chose EfficientNet B2 to replace Darknet53 as the backbone for feature extraction. An FPN structure was used for the fusion of feature layers to combine different information about the features. Three effective feature layers of different sizes obtained by EfficientNet B2 and FPN were regressed and classified by YOLO Head. **(B)** CNN2 is based on UNet. To obtain better feature extraction, we used pretrained VGG-16 instead of UNet’s backbone (left). After a series of upsampling and concatenation operations, the 5 effective feature layers extracted by VGG-16 were dimensionally superimposed and convolved twice (right). Finally, the final segmentation result was output after a softmax function. CNN, convolutional neural network; FPN, feature pyramid networks.

The CAD system consists of CNN1, which is responsible for diagnosis, and CNN2, which is responsible for the lesion segmentation and localisation. CNN1 is based on YOLO V3 (19). EfficientNet B2 was used as the backbone to replace the original DarkNet53 to obtain better feature extraction results (**Figure 3A**). The EfficientNet B2 model was pretrained on the Pascal VOC 2007 dataset and used as the initial weights. CNN2 is based on UNet, and a pretrained VGG-16 on ImageNet was used to replace the backbone of UNet (**Figure 3B**) (20, 21). Feature pyramid networks (FPN) (22) and an attention module (23) were additionally added to improve the feature extraction performance.

To improve the accuracy and the generalisation and to avoid overfitting, the shallow-level convolutional layers of the pretrained DarkNet 53 and VGG-16 were frozen, and the weights of the high-level convolutional layers were updated during the training. The initial image size was 416×416 pixels, 8 images were put into training at a time, and the learning rate was automatically adjusted for the rate of decline. The model was evaluated every 10 iterations to calculate the average accuracy of the validation set and save the weights. The total number of training iterations was 500 in CNN1 and 100 in CNN2. Training details for CNN1 and CNN2 are provided in the **Supplementary Material**.

### 2.4 Evaluation of the deep learning-based CAD system

In the CNN1 model, the Youden index was used to calculate the threshold for binary classification. If the output confidence value of a neoplastic image is higher than the specified threshold, the image is considered a true positive (TP), and if it is less than the specified threshold, it is considered a false negative (FN). A non-neoplastic image is considered a false positive (FP) if its confidence value is higher than the specified threshold or a true negative (TN) if it is less than the specified threshold.

The diagnostic performance of CNN1 was evaluated by accuracy, sensitivity, specificity, positive predictive value (PPV), negative predictive value (NPV), receiver operating characteristic (ROC) curve and area under the curve (AUC).


$$accuracy=\frac{TP+TN}{TP+FP+TN+FN}$$

In the CNN2 model, intersection over union (IOU), precision, recall and the Dice coefficient were used to evaluate the segmentation performance. IOU means the ratio of the intersection and union of the AI segmentation region to the lesion labelled by an expert endoscopist (ground truth). If the IOU between the CNN2 segmentation and the ground truth is higher than a specified threshold, it is considered a TP; otherwise, it is an FP. If IOU = 0, it is considered an FN.


$$IOU=\frac{\left|X\cap^{\;}Y\right|}{\left|X\right|\cup^{\;}\left|Y\right|}$$

Precision, recall, and the Dice coefficients (24) were calculated as follows:


$$precision=\frac{TP}{TP+FP}$$

$$recall=\frac{TP}{TP+FN}$$

$$Dice=\frac{2*\left|X\cap^{\;}Y\right|}{\left|X\right|\cup^{\;}\left|Y\right|}=\frac{2TP}{2TP+FP+FN}$$

To test for differences in the diagnostic outcomes between the CAD system and the endoscopists, endoscopists of different experience levels were selected to judge the images in the test set. Three endoscopists were considered senior endoscopists with more than five years of endoscopy experience. Moreover, they have received education and training in identifying EGC under ME-NBI and had participated in EGC screening. Three endoscopists were regarded as junior endoscopists with less than five years of experience in endoscopy and no specific EGC training.

### 2.5 Statistical analysis

All statistical analyses were conducted using SPSS 22.0 (IBM, Armonk, NY, USA). The 95% confidence intervals (95% CI) were evaluated using the Wilson method. Continuous variables were compared by one-way ANOVA, and categorical variables were compared by the chi-square test or Fisher’s exact test. A chi-square test was conducted to compare the accuracy, sensitivity, specificity, PPV and NPV between CNN1 and the endoscopists. A p value less than 0.05 was considered as statistically significant.

## 3 Results

### 3.1 Clinicopathological characteristics of the patients and lesions

This study included 498 patients diagnosed with GNLs, including LGIN (n=296) and EGC (n=202), who were separated into a training set (n=333), internal test set (n=59) and external test set (n=106). The characteristics of the patients and lesions from the three sets are shown in **Table 1**. Patients in all three datasets showed no significant differences in terms of mean age, gender composition, lesion location or macroscopic type, except for a higher proportion of patients with EGC in the external test set. For EGC lesions, there were no significant differences in the mean diameter, depth of infiltration and degree of differentiation among patients from the three sets.

**Table 1:** Clinical characteristics of patients in the training set and test sets.

	Training Set	Internal Test Set	External Test Set	*p-*value
**Patients**	333	59	106	
**Age, average ± SD, (range), years**	64.1 ± 11.0 (28-87)	64.0 ± 11.0 (31-82)	62.9 ± 11.1 (26-86)	0.800
**Sex, no. (%)**				0.622
Male	214 (64.3%)	34 (57.6%)	67 (63.2%)	
Female	119 (35.7%)	25 (42.4%)	39 (36.8%)	
**Lesion location, no. (%)**				0.180
Upper third	35 (10.5%)	7 (11.9%)	9 (8.5%)	
Middle third	72 (21.6%)	20 (33.9%)	31 (29.2%)	
Lower third	226 (67.9%)	32 (54.2%)	66 (62.3%)	
**Macroscopic types‡, no. (%)**				0.345
0-IIa	112 (33.6%)	17 (28.8%)	32 (30.2%)	
0-IIb	48 (14.4%)	8 (13.6%)	15 (14.2%)	
0-IIc	116 (34.8%)	19 (32.2%)	30 (28.3%)	
0-I	5 (1.5%)	0	1 (0.9%)	
0-III	2 (0.6%)	1 (1.7%)	0	
Mix Type	50 (15%)	14 (23.7%)	28 (26.4%)	
**Vienna Classification, no. (%)**				<0.001
Category 3 (LGIN)	216 (64.9%)	39 (66.1%)	41 (38.7%)	
Category 4 and 5 (EGC)	117 (35.1%)	20 (33.9%)	65 (61.3%)	
**Invasion depth of EGC, no. (%)**				0.673
Intraepithelial neoplasia	50 (42.7%)	8 (40.0%)	21 (32.3%)	
Intramucosal carcinoma	50 (42.7%)	10 (50.0%)	33 (50.8%)	
Submucosal carcinoma	17 (14.5%)	2 (10.0%)	11 (16.9%)	
**Diameter of EGC, average ± SD, range, cm**	1.69 ± 1.15 (0.2-6.5)	2.01 ± 1.75 (0.4-7.5)	1.79 ± 1.06 (0.2-5)	0.542
**Histologic type of EGC‡‡, no. (%)**				0.857
Differentiated type	87 (74.4%)	15 (75.0%)	46 (70.8%)	
Undifferentiated type	30 (25.6%)	5 (25.0%)	19 (29.2%)	

^‡^ According to the Paris endoscopic classification. 0-IIa, flatly elevated; 0-IIb, flat; 0-IIc, flatly depressed; mix type, 0-IIa+IIc, 0-IIc+IIa, 0-IIb+IIc and 0-IIc+IIb; 0-I, protruded; 0-III, excavated.

^‡‡^ According to the Nakamura classification.

SD, standard deviation; LGIN, low-grade intraepithelial neoplasia; EGC, early gastric cancer.

### 3.2 Diagnostic performance of CNN1

**Figure 4:**
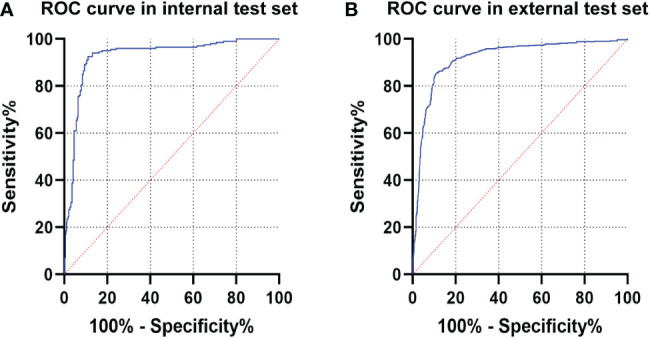
The ROC curve of CNN1 in the test sets. **(A)** ROC curve of CNN1 in the internal test set. **(B)** ROC curve of CNN1 in the external test set. ROC, receiver operating characteristic; CNN, convolutional neural network.

The diagnostic performance of CNN1 was tested in the internal and external test sets. The maximum Youden index is achieved when the classification threshold is set to 0.4920. In the internal test set, CNN1 correctly diagnosed 363 of 400 ME-NBI images with an AUC of 0.928 (95% CI, 0.899-0.956) and an accuracy of 90.8% (95%CI, 87.9%-93.6%). In the external test set, CNN1 diagnosed 1372 of 1600 ME-NBI images with an AUC of 0.918 (95% CI, 0.903-0.932) and an accuracy of 85.8% (95%CI, 84.0%-87.5%). The diagnostic performance of CNN1 is shown in **Table 2**, and the ROC curves of the internal and external test sets are shown in **Figures 4A, B**.

**Table 2:** Diagnostic performance of CNN1 in internal and external test sets.

	Internal Test Set		External Test Set	
**Accuracy**	363/400	90.8% (87.9%-93.6%)	1372/1600	85.8% (84.0%-87.5%)
**Sensitivity**	185/200	92.5% (88.8%-96.2%)	701/800	87.6% (85.3%-89.9%)
**Specificity**	178/200	89.0% (84.6%-93.4%)	671/800	83.9% (81.3%-86.4%)
**PPV**	185/207	89.4% (85.1%-93.6%)	701/830	84.5% (82.0%-86.9%)
**NPV**	178/193	92.2% (88.4%-96.0%)	671/770	87.1% (84.8%-89.5%)
**AUC**	0.928 (0.899-0.956)		0.918 (0.903-0.932)	

CNN, convolutional neural network; PPV, positive predictive value; NPV, negative predictive value; AUC, area under the curve.

### 3.3 Comparison of the diagnostic performance between CNN1 and the endoscopists

To compare the diagnostic performance between CNN1 and the endoscopists, six endoscopists (three senior endoscopists and three junior endoscopists) who had not participated in the previous work on this study were selected to analyse the 100 images in the comparison test set. The diagnostic performance of GNLs by CNN1 and the endoscopists is shown in **Table 3**. The data showed that CNN1 outperformed the senior and junior endoscopists in average diagnostic performance.

**Table 3:** Comparison between CNN1 and endoscopists in the diagnosis of GNLs.

	Accuracy	Sensitivity	Specificity	PPV	NPV
**CNN1**	88.0% (81.5%-94.5%)	90.0% (81.4%-98.6%)	86.0% (76.0%-96.0%)	86.5% (76.9%-96.1%)	89.6% (80.6%-98.5%)
**Senior endoscopists (n=3)**	72.7%** (67.6%-77.7%)	76.0%* (69.1%-82.9%)	69.3%* (61.9%-76.8%)	71.3%* (64.2%-78.3%)	74.3%* (67.0%-81.6%)
**Junior endoscopists (n=3)**	62.7%** (57.2%-68.2%)	59.3%** (51.4%-67.3%)	66.0%** (58.3%-73.7%)	63.6%** (55.5%-71.6%)	61.9%** (54.3%-69.5%)

**p<0.01, *p<0.05 (vs. CNN1); CNN, convolutional neural network; GNLs, gastric neoplastic lesions; PPV, positive predictive value; NPV, negative predictive value.

**Figure 5:**
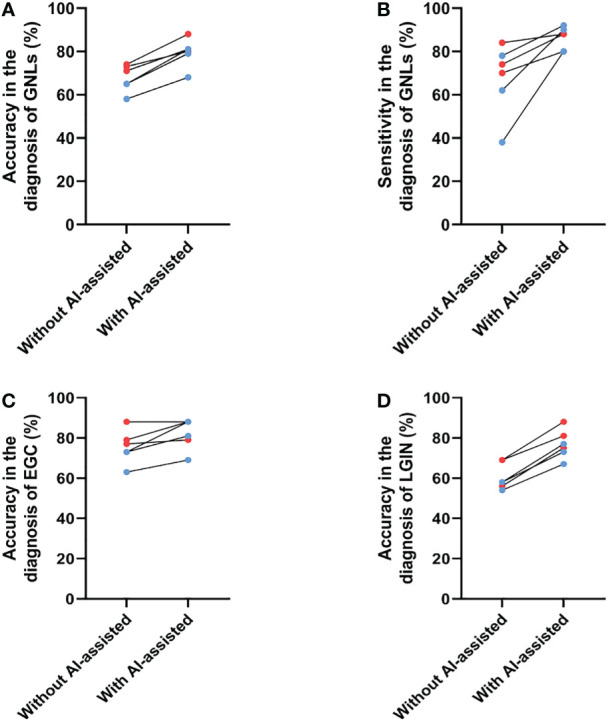
Changes in the diagnostic performance for GNLs of endoscopists before and after CNN1 assistance. **(A)** Changes in accuracy for GNLs of endoscopists before and after CNN1 assistance. **(B)** Changes in sensitivity for GNLs of endoscopists before and after CNN1 assistance. **(C)** Changes in the accuracy for EGC of endoscopists before and after CNN1 assistance. **(D)** Changes in the accuracy for LGIN of endoscopists before and after CNN1 assistance. Red dots represent senior endoscopists, and blue dots represent junior endoscopists. AI, artificial intelligence; GNLs, gastric neoplastic lesions; CNN, convolutional neural network; EGC, early gastric cancer; LGIN, low-grade intraepithelial neoplasia.

Next, the performance of the endoscopists in diagnosing GNLs with CNN1 assistance was further evaluated. The endoscopists rediagnosed the same images with CNN1 assistance at least two weeks after their previous independent diagnosis. The results showed an average increase in diagnostic accuracy of 11.8%, with an average improvement of 10.3% for senior endoscopists and an average of 13.3% for junior endoscopists (**Figure 5A**). Because of differences in their diagnostic style, the improvement in sensitivity after CNN1 assistance varied considerably among the endoscopists, with an average improvement in sensitivity of 18.7%, including 9.3% for senior endoscopists and 28.0% for junior endoscopists (**Figure 5B**). The diagnostic performance of each endoscopist for GNLs is shown in **Table 4**.

**Table 4:** Diagnostic performance for GNLs by each endoscopist.

	Accuracy	Sensitivity	Specificity	PPV	NPV
**CNN1**	0.880 (0.815-0.945)	0.900 (0.814-0.986)	0.860 (0.760-0.960)	0.865 (0.769-0.961)	0.896 (0.806-0.985)
**Senior 1**	0.740* (0.653-0.827)	0.740* (0.614-0.866)	0.740 (0.614-0.866)	0.740 (0.614-0.866)	0.740* (0.614-0.866)
**Senior 2**	0.710** (0.620-0.800)	0.700* (0.568-0.832)	0.720 (0.591-0.849)	0.714 (0.583-0.845)	0.706* (0.576-0.835)
**Senior 3**	0.730** (0.641-0.819)	0.840 (0.735-0.945)	0.620** (0.481-0.759)	0.689* (0.569-0.808)	0.795 (0.662-0.927)
**Junior 1**	0.580** (0.482-0.678)	0.620** (0.481-0.759)	0.540** (0.397-0.683)	0.574** (0.438-0.710)	0.587** (0.439-0.735)
**Junior 2**	0.650** (0.555-0.745)	0.380** (0.241-0.519)	0.920 (0.842-0.998)	0.826 (0.658-0.994)	0.597** (0.485-0.709)
**Junior 3**	0.650** (0.555-0.745)	0.780 (0.661-0.899)	0.520** (0.377-0.663)	0.619** (0.496-0.742)	0.703* (0.548-0.857)

"**p<0.01, *p<0.05 (vs. CNN1); GNLs, gastric neoplastic lesions; CNN, convolutional neural network; PPV, positive predictive value; NPV, negative predictive value.

Specifically, the average accuracy of diagnosing EGC increased 3.5% among the senior endoscopists, which may be because the participating senior endoscopists were already very good at diagnosing EGC under ME-NBI, and it increased 9.7% among the junior endoscopists (**Figure 5C**, **Table 5**). The use of CNN1 significantly improved the specificity of EGC diagnosis by senior endoscopists (68.1% to 80.6%) and the sensitivity of EGC diagnosis by junior endoscopists (75.0% to 91.7%) (**Table 5**). For LGIN lesions, the average accuracy was significantly increased for both senior and junior endoscopists (both at 16.7%) (**Figure 5D**, **Table 6**). This suggests that with CNN1 assistance, the endoscopist’s ability to diagnose LGIN is greatly improved and will reduce the rate of missed LGIN diagnoses.

**Table 5:** Comparison between CNN1 and endoscopists for the diagnosis of EGC.

		Accuracy	Sensitivity	Specificity	PPV	NPV
**CNN1**		89.6% (80.6%-98.5%)	91.7% (79.7%-100%)	87.5% (73.2%-100%)	88.0% (74.3%-100%)	91.3% (78.8%-100%)
**Senior endoscopists** **(n=3)**	**without CNN1-assistance**	81.3% (74.8%-87.7%)	94.4% (89.0%-99.9%)	68.1% (57.0%-79.1%)	74.7% (65.6%-83.8%)	92.5% (85.1%-99.8%)
	**with CNN1-assistance**	84.7% (78.8%-90.7%)	88.9% (81.5%-96.3%)	80.6% (71.2%-89.9%)	82.1% (73.3%-90.8%)	87.9% (79.8%-96.0%)
**Junior endoscopists** **(n=3)**	**without CNN1-assistance**	69.4% (61.8%-77.1%)	75.0% (64.8%-85.2%)	63.9% (52.5%-75.3%)	67.5% (57.0%-78.0%)	71.9% (60.6%-83.2%)
	**with CNN1-assistance**	79.2% (72.5%-85.9%)	91.7% (85.1%-98.2%)	66.7% (55.5%-77.8%)	73.3% (64.0%-82.6%)	88.9% (80.2%-97.5%)

CNN, convolutional neural network; EGC, early gastric cancer; PPV, positive predictive value; NPV, negative predictive value.

**Table 6:** Comparison between CNN1 and endoscopists for the diagnosis of LGIN.

		Accuracy	Sensitivity	Specificity	PPV	NPV
**CNN1**		86.5% (76.9%-96.1%)	88.5% (75.3%-100%)	84.6% (69.8%-99.5%)	85.2% (70.9%-99.5%)	88.0% (74.3%-100%)
**Senior endoscopists** **(n=3)**	**without CNN1-assistance**	64.7% (57.2%-72.3%)	59.0% (47.8%-70.1%)	70.5% (60.2%-80.9%)	66.7% (55.3%-78.1%)	63.2% (52.9%-73.6%)
	**with CNN1-assistance**	81.4% (75.2%-87.6%)	82.1% (73.3%-90.8%)	80.8% (71.8%-89.7%)	81.0% (72.2%-89.9%)	81.8% (73.0%-90.6%)
**Junior endoscopists** **(n=3)**	**without CNN1-assistance**	56.4% (48.5%-64.3%)	44.9% (33.6%-56.2%)	67.9% (57.4%-78.5%)	58.3% (45.5%-71.2%)	55.2% (45.1%-65.3%)
	**with CNN1-assistance**	73.1% (66.0%-80.1%)	83.3% (74.9%-91.8%)	62.8% (51.9%-73.8%)	69.1% (59.6%-78.7%)	79.0% (68.6%-89.5%)

CNN, convolutional neural network; LGIN, low-grade intraepithelial neoplasia; PPV, positive predictive value; NPV, negative predictive value.

### 3.4 Segmentation performance of CNN2

**Figure 6:**
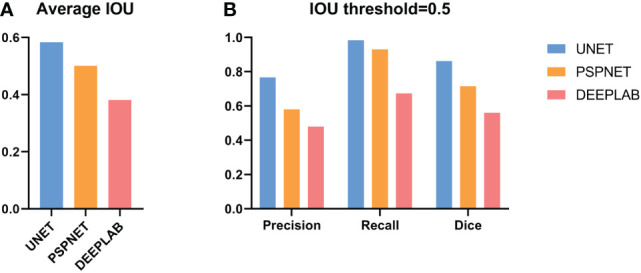
Performance of CNN2 and other deep models on the test set. **(A)** The average IOU compared between UNet, PSPNet and DeepLab. **(B)** The precision, recall and the Dice coefficient of UNet, PSPNet and DeepLab at the IOU threshold of 0.5. CNN, convolutional neural network. IOU, Intersection over union.

**Figure 7:**
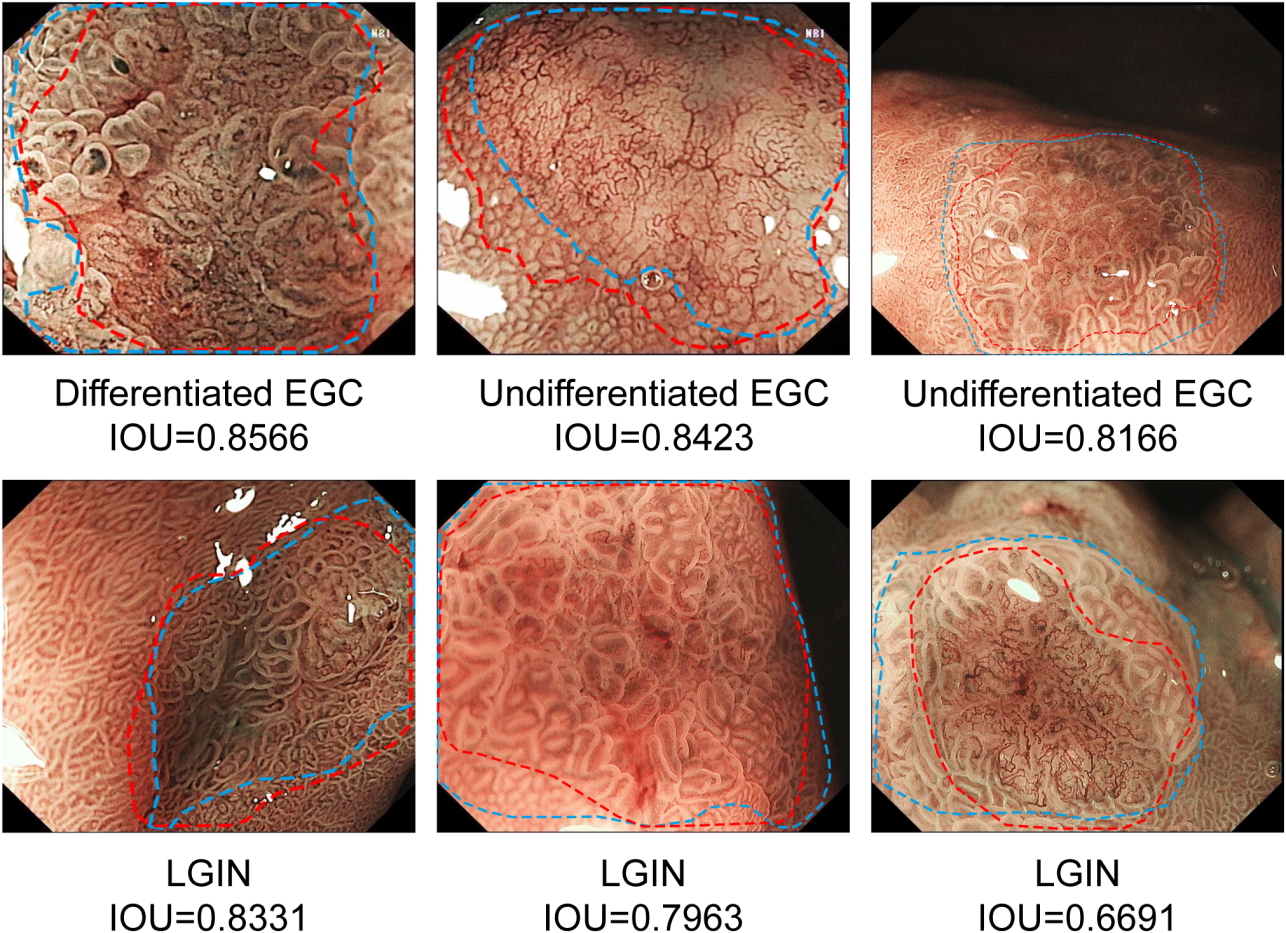
Examples of segmentation performance of CNN2 in the test sets. The margins of the neoplastic lesions labelled by an expert endoscopist are shown as blue lines, and the margins of the lesions predicted by CNN2 are shown as red lines. CNN, convolutional neural network.

The results show that CNN2 has a better segmentation effect on GNLs. The average IOU between CNN2 and the expert label was 0.584. To assist in the endoscopic resection of GNLs, the IOU threshold was set at 0.5. The precision, recall and Dice coefficient were 0.776, 0.983 and 0.867, respectively. The performance of CNN2 and other segmentation modules was tested to compare the segmentation effect. CNN2 exhibits better segmentation than PSPNET and DEEPLAB in average IOU, precision, recall and Dice coefficient (**Figure 6**). The segmentation performance of different GNLs is shown in **Figure 7**.

## 4 Discussion

In this study, a CAD system based on deep learning was developed for the diagnosis and segmentation of GNLs under ME-NBI. The CAD system achieves good diagnostic performance, with an accuracy, sensitivity, specificity, NPV and PPV of the system for diagnosing GNLs in the internal test set of 90.8%, 92.5%, 89.0%, 89.4% and 92.2%, respectively, and it was subjected to external testing. When the IOU threshold was set at 0.5, CNN2 showed a precision, recall and the Dice coefficient of 0.776, 0.983 and 0.867, respectively.

ESD has been widely used in the treatment of EGC and HGIN because of its low invasiveness and low risk of lymph node metastasis; however, whether LGIN lesions should be treated with endoscopic resection remains controversial (25–28). It has been reported that 15-26.9% of LGIN lesions develop into HGIN or gastric cancer (29, 30). In addition, for LGIN lesions, the overall discrepancy rate of pathological results between biopsy specimens and ESD specimens is high, and approximately 24% of lesions show elevated histological upstaging after ESD (31, 32). Depressed morphology, largest diameter of the lesion, surface unevenness and surface erythema had a higher odds ratio with potential histological upstaging (28, 32–34). Therefore, the latest Western guidelines recommend endoscopic resection of both endoscopically visible neoplastic lesions, including LGIN, to reduce the risk of malignant transformation (4).

The application of ME-NBI has significantly improved the diagnosis rate of superficial GNLs (35, 36). However, there are still some limitations that affect its clinical application. There is currently a well-established system for diagnosing EGC under ME-NBI, but endoscopists need to be well educated in ME-NBI. In addition, atypical LGIN is easily missed during an endoscopic examination because the diversity of lesions often makes it difficult to distinguish LGIN lesions from other non-neoplastic mucosa. Therefore, a deep-learning based CNN1 was developed to diagnose GNLs under ME-NBI. CNN1 showed a great performance in both the internal test set and the external test set.

In this study, the accuracy of senior endoscopists in diagnosing EGC was close to that of CNN1 in our test (**Table 5**). However, for all of the endoscopists tested, including senior and junior endoscopists, their diagnostic performance for diagnosing LGIN lesions was poor with a high rate of missed diagnosis (**Table 6**). With the assistance of CNN1, senior endoscopists had improved accuracy and sensitivity for diagnosing LGIN without reducing their diagnostic performance for EGC, while junior endoscopists had improved the diagnostic ability for both EGC and LGIN lesions. All endoscopists who participated in the test reported more confidence in their diagnosis of GNLs with the assistance of CNN1, especially for LGIN lesions. Moreover, all three junior endoscopists believed that the auxiliary value of CNN1 was primarily reflected in which lesions needed targeted biopsy for patients with multiple suspected gastric lesions.

**Figure 8:**
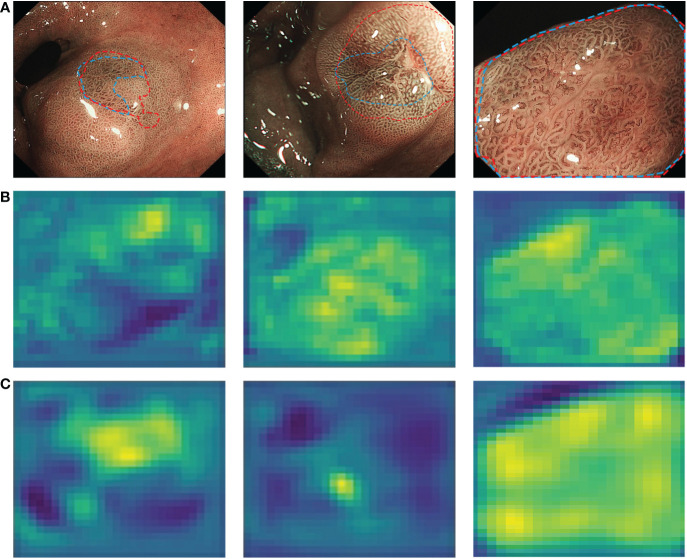
Characteristic layers extracted from endoscopist-labelled ME-NBI images by CNN2. **(A)** The margins of neoplastic lesions labelled by an expert endoscopist are shown as blue lines, and the margins predicted by CNN2 are shown as red lines. **(B)** Shallow characteristic layers of vascular and texture details extracted by CNN2. **(C)** Deep characteristic layers of colour details extracted by CNN2. A lighter colour indicates a high possibility of an abnormal area. ME-NBI, magnifying endoscopy with narrow-band imaging; CNN, convolutional neural network.

In addition to correct diagnosis, accurate delineation of the lesion margins is necessary for accurate lesion biopsy and endoscopic resection (37). However, this requires sufficient clinical practice and experience and is difficult for junior endoscopists. To address this problem, a semantic segmentation-based CNN2 was developed to depict of the margins of GNLs. A preliminary endoscopic diagnosis of suspicious lesions was performed based on the characteristics of LGIN and EGC under ME-NBI as described in previous studies (6, 8–11) and endoscopic experts labelled the extent of the lesions based on the pathology data. Combining the feature extraction network VGG-16 with the improved UNet structure, CNN2 can effectively extract the vascular and texture details of the shallow feature layers and the colour details of the deep feature layers from the lesion images, which can perform better in lesion segmentation. **Figure 8** shows the features extracted by CNN2. When the IOU threshold was set to 0.5, CNN2 showed a recall score of 0.983, which means that it can be used as a complement to the CNN1 diagnostic results to maximise the identification of the potential GNLs. In addition, the precision of 0.776 assists endoscopists in determining the margins of GNLs during endoscopic biopsy or ESD treatment, thus improving the accuracy of the biopsy or the complete resection rate of the lesions and reducing the risk of positive horizontal resection margins in pathological evaluation after ESD.

The advantages of our study are as follows: first, this study is a multicenter study, and images from three centers were used for training and testing. Second, the CAD system was trained and tested based on GNLs for the first time. Third, this CAD system combines two CNN modules to enable the diagnosis and segmentation of neoplastic lesions and it showed great performance.

However, there are still some defects in this CAD system. First, due to technical limitations, this model is still an “offline” system, which only allows the input of ME-NBI images for diagnosis and segmentation. This problem can be addressed by improving the data transmission and analysis speed, or by using other techniques to obtain the results of the AI analysis during endoscope examination to guide the diagnosis and treatment in real time. Second, this model cannot output the interpretability of the model yet. Futher studies will improve the performance of this model by adding a heatmap module. Third, low-quality images, such as bleeding and mucus coverage, were excluded during the training. However, these images may also have some value in assisting endoscopists with the diagnosis. In future studies, these images will be incorporated to achieve a generalisation of the model.

In summary, the newly developed CAD system can be used as a high-sensitivity auxiliary tool to diagnose and determine the margins of GNLs under ME-NBI in patients with suspected superficial lesions. This system can assist endoscopists in identifying neoplastic lesions quickly and accurately by marking the margins of the lesions to assist them in making correct decisions and achieving precise treatment. This will ultimately reduce the risk of progression to gastric cancer and avoid the serious consequences of gastric cancer.

## Data availability statement

The raw data supporting the conclusions of this article will be made available by the authors, without undue reservation.

## Author contributions

Conceptualization: HZ, XJW, and LGL; Methodology: LHL, JC, ZD, WN, JX, TM, and XWW; Formal analysis and investigation: LHL, XB, KQ, CY, JW, and WN; Writing - original draft preparation: LHL, JC, and ZD; Writing - review and editing: HZ, XJW, and LGL; Funding acquisition: HZ and XJW; Resources: HZ, XJW, LGL, and XB; Supervision: HZ, XB, and JW. All authors contributed to the article and approved the submitted version.
