# Supplementary material for: Diagnosis and segmentation effect of the ME-NBI-based deep learning model on gastric neoplasms in patients with suspected superficial lesions - a multicenter study
Source: Front Oncol. 2023 Jan 16;12:1075578. doi: 10.3389/fonc.2022.1075578 (PMC9885211; doi:10.3389/fonc.2022.1075578)
Supplement: Supplementary file 1 [file DataSheet_1.pdf]

Appendix

1.1 System configuration of training CNN1 and CNN2：

In this study, CNN1 and CNN2 were developed based on Python 3.9 and PyCharm in Ubuntu 18.04 system. Models were implemented with Tensorflow 2.5 and Keras. Training was accomplished with a NVIDIA GeForce RTX 3090Ti GPU.

1.2 Details of CNN1 training

CNN1 was based on YOLO V3 and EfficientNet B2 was used to replace the backbone Darknet 53. Pretrained EfficientNetB2-YOLOv3 model parameters on the Pascal VOC 2007 dataset were used as initial weights. The model had 401 layers with approximately 1566 million parameters. We froze the pre-trained feature extraction part of EfficientNetB2, the shallower 333 layers, and updated the weights of the higher convolutional layers during the training process to improve accuracy and generalization and avoid overfitting problems.

To match the fully connected layer of YOLO V3, the input images were resized to 416 x 416 pixels. The batch size and epoch was set to 8 and 200, respectively. 8 images were put into training at a time. The Adam optimizer was employed to update the parameters until convergence [1]. The learning rate was initialized at 0.0001 and the learning rate was automatically adjusted for the rate of decline. The dropout rate was set to 0.5. The model was evaluated every 10 iterations to calculate the average accuracy of the validation set and save the weights. If the accuracy was currently optimal, it was considered as best epoch weights. The total number of training iterations was 500 in CNN1.

The loss function was the same as the original loss function of YOLO V3 model. In short, the loss function of the model was the sum of three loss functions: bounding box prediction (L_{box}), object prediction (L_{obj}) and classification prediction (L_{cls}) [2].

$$L_{total} = L_{box} + L_{obj} + L_{cls}$$

1.3 Details of CNN2 training

CNN2 was developed based on UNet [3]. This network mainly includes a encoder structure, a decoder sturcture and jump connection components. A VGG-16 model was used to replace the feature extraction network of UNet and its last full connection layer was removed. Before training, VGG-16 was pretrained on ImageNet [4]. Based on the pretrained weight of VGG16 feature extraction network on ImageNet, we frozen some shallow networks and fine-turned the back-end parameters of the network to avoid the over fitting problem, which played a role in improving the final accuracy and generalization of the model.

In order to obtain a better feature extraction effect, we added a feature pyramid network (FPN) structure [5]. FPN can link the deep features of low-resolution and

high semantic information with shallow features of high-resolution and low semantic information from top to bottom. Therefore, it can make the features of different scales contain rich semantic information.

Image of size (448,448,3) was input to VGG16. After the convolution layer with a two-layer convolution kernel of 3×3 and filter of 64, the first feature layer P1 (448,448,64) was output. Then, the first feature layer was pooled with a step size of 2, and the convolution layer with a filter of 128 and a convolution kernel of 3×3 was also carried out twice. At this time, the output feature layer was denoted as P2 (224,224,128). Similarly, we continue to pool and convolute P2. The difference was that the next three and four layers, namely P3 and P4, had three convolution operations after pooling. Finally, after pooling the fourth layer of feature extraction layer and three times of convolution, the output of the last layer of feature layer, namely P5, can be obtained. The convolution operation with filter 256 was carried out for each feature layer, which can make the convolution feature dimension of the deep layer be the same as that of the adjacent shallow layer. After the above operations were completed, the upsampling operation was carried out on the deep convolution results. At this time, the size of the output feature layer will be the same as that of the adjacent shallow feature layer. Through addition operation, more color and texture information contained in the shallow layer will be supplemented in corresponding details to the deep layer, making the inclusion information of the fused feature layer more abundant. Finally, the feature layer obtained by P5 convolution was convolved with filter 256 as the feature5 output by UNet coding part. In addition, take the same convolution operation on the feature layer after each add operation, so that the remaining four feature layers can be obtained as the feature extraction input of the UNet coding layer.

In addition, an attentional mechanism was added to CNN2 [6]. The basic idea of attentional mechanism was to focus more attention on the regions with important features by suppressing the background information, so as to improve the feature expression ability and attention degree of the region of interest. The first four feature layers of the pyramid output were globally averaged pooled to obtain a one-dimensional matrix whose length was the same as the number of feature layer channels in the original input. Secondly, take two full connection operations on the matrix, so as to achieve the narrowing and recovery of the channel. The one-dimensional matrix parameters obtained by this method can be used as weight parameters to multiply each layer channel. The above operations reduced the loss of detail features and small objects in the subsampling process, thus realizing the attention mechanism in the convolutional neural network. Then, channel superposition and convolution operation of UNet decoding layer were carried out with the feature layer obtained from the corresponding up-sampling after integrating the attention mechanism. Finally, the final output of the model can be obtained by normalization.

The size of the input images was adjusted to 448 × 448 pixels, and four images were put into training each time. The batch size was 8. The total number of training iterations was 100. After comparison, we chose to use the initial learning rate of e-4

for the first 50 freezing iterations and the learning rate of e-5 for the next 50 iterations. The Adam optimizer was selected, and the dropout rate was set at 0.5.

The combined function of cross-entropy (CE) and Dice loss was chosen as the loss function for CNN2. In our preliminary experiments, this combined loss function showed better performance to other loss functions. The loss functions were calculated as follows:

$$\mathrm{L_{DiceCE}} = \mathrm{L_{CE}} + \mathrm{L_{Dice}}$$

$$\mathrm{L_{CE}} = -\sum_{i=0}^{n} p(i)q(i)$$

$$\mathrm{L_{Dice}} = \frac{2|\mathrm{X} \cap \mathrm{Y}|}{|\mathrm{X}| \cup |\mathrm{Y}|}$$

References

[1] Kingma D, Ba J. Adam: A Method for Stochastic Optimization; 2014. arXiv Preprint arXiv:1412.6980v9.

[2] Redmonm J, Farhadi A. Yolov3: An Incremental Improvement; 2018. arXiv Preprint, arXiv:1804.02767.

[3] Ronneberger O, Fischer P, Brox T. U-net: convolutional networks for biomedical image segmentation, International Conference on Medical Image Computing and Computer-Assisted Intervention 2015: Medical Image Computing and Computer-Assisted Intervention-MICCAI 2015, 234–41.

[4] Simonyan K, Zisserman A. Very deep convolutional networks for large-scale image recognition; 2014. arXiv Preprint arXiv:1409.1556v6.

[5] Lin TY, Dollar P, Girshick R, He K, Hariharan B, Belongie S. Feature Pyramid Networks for Object Detection; 2016. arXiv Preprint arXiv:1612.03144.

[6] Bahdanau D, Cho K, Bengio Y. Neural Machine Translation by Jointly Learning to Align and Translate; 2014. arXiv Preprint arXiv:1409.0473.

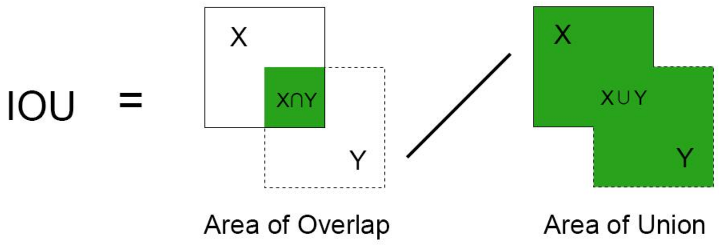

Fig. 1 Visual description of IOU. The IOU is calculated by dividing the area of overlap by the area of union for both subsets. IOU, intersection over union.
